# Knowledge, Attitude and Perception of Research Ethics and Research Ethics Committees among Post-Graduate Residents of Neurosciences - A Nationwide Analysis from Pakistan

**DOI:** 10.12669/pjms.40.12(PINS).11116

**Published:** 2024-12

**Authors:** Haseeb Mehmood Qadri, Raana Shahid, Shahar Bano Fatima, Neha Naveed, Ahtesham Khizar, Siraj ul Haq, Zubair Mustafa Khan, Asif Bashir, Group Collaborator

**Affiliations:** 1 Dr. Haseeb Mehmood Qadri, MBBS, Post Graduate Resident, Department of Neurosurgery, Punjab Institute of Neurosciences, Lahore, Pakistan; 2 Dr. Raana Shahid, MBBS, Post Graduate Resident, Department of Neurosurgery, Punjab Institute of Neurosciences, Lahore, Pakistan; 3 Dr. Shahar Bano Fatima, MBBS, Post Graduate Resident, Department of Neurosurgery, Punjab Institute of Neurosciences, Lahore, Pakistan; 4 Dr. Neha Naveed, MBBS, Medical Officer, Department of Neurosurgery, Combined Military Hospital, Muzaffarabad, Azad Kashmir, Pakistan; 5 Dr. Ahtesham Khizar, MBBS, Senior Registrar, Department of Neurosurgery, Department of Neurosurgery, Punjab Institute of Neurosciences, Lahore, Pakistan; 6 Dr. Siraj ul Haq, MBBS, Post Graduate Resident, Department of Neurosurgery, Rehman Medical Institute, Peshawar, Pakistan; 7 Dr. Zubair Mustafa Khan, MBBS, Assistant Professor, Department of Neurosurgery, Punjab Institute of Neurosciences, Lahore, Pakistan; 8 Prof. Dr. Asif Bashir, MBBS, Diplomat American Board of Neurosurgery, Department of Neurosurgery, Punjab Institute of Neurosciences, Lahore, Pakistan; 9 Collaborator Group, Dr. Arham Amir Khawaja, *MBBS*, Department of General Surgery, Sheikh Zayed Hospital, Lahore, Pakistan

**Keywords:** Knowledge, Attitude, Research Ethics, Research Ethics Committees, Bioethics, Pakistan

## Abstract

**Objectives:**

To assess the knowledge, attitude and perception of post-graduate residents of neurosciences towards research ethics (RE) and research ethics committees (RECs).

**Methods:**

This prospective cross-sectional survey-based study was conducted by the Punjab Institute of Neurosciences, targeting post-graduate neuroscience residents throughout Pakistan during January and February, 2024. An English-language general questionnaire, designed to assess knowledge, attitudes, and perceptions of RE and RECs, was adapted to reflect local conditions. The collected responses were analyzed using the Statistical Package for Social Sciences (SPSS) to determine measures of central tendency, percentages, and frequencies.

**Results:**

Out of 241 residents, 64.3% were males. Sixty-eight percent respondents were from neurosurgery and 32% were from the field of neurology. Approximately 47% responses were from the province of Punjab. More than half (51.5%) of participants acknowledged the existence of a REC within their institution. A similar proportion (52.3%) claimed familiarity with ethical guidelines for research involving human subjects, only a slight majority (48.5%) demonstrated awareness of the specific functions carried out by RECs. Meanwhile, 44.5% expressed concerns that undergoing review by a REC could potentially delay research and pose additional challenges for researchers. Additionally, 27.4% of respondents admitted to considering the fabrication of data or results as acceptable.

**Conclusion:**

Our research uncovered a significant correlation between participants’ perceptions of RE and RECs and their comprehension of ethical principles. These findings indicate that medical postgraduates with a deeper understanding or awareness of research ethics principles and RECs tend to hold more robust attitudes toward these aspects.

**List of Abbreviations:**
AAHRPP: Association for the Accreditation of Human Research Protection Program,
DME: Department of Medical Education,
DRAP-CSC: Drug Regulatory Authority of Pakistan-Clinical Studies Committee,
FCPS: Fellow of College of Physicians and Surgeons,
IRBs: Institutional Review Boards,
LMICs: Low-Middle-Income Countries,
MCQs: Multiple Choice Questions,
MD: Master of Medicine,
MS: Master of Surgery,
NBC-REC: National Bioethics Committee-Research Ethics Committee,
PMDC: Pakistan Medical and Dental Council,
RE: Research Ethics,
RECs: Research Ethics Committees,
SPSS: Statistical Package for Social Sciences

## INTRODUCTION

Biomedical ethics is the study of moral principles and values that inform ethical decision-making in healthcare, medical research, and related disciplines. Research ethics (RE) are guidelines that ensure research is conducted with transparency, integrity and respect. They also safeguard the rights and welfare of all involved subjects including humans and animals while maintaining the authenticity of the collected data and the accuracy of the findings presented. The roots of biomedical ethics can be traced back to the inception of the Hippocratic Oath.^1^ In the past few decades, it has evolved into an academic interdisciplinary field. With recent global advancement of educational standards significant transformations have occurred in medical education, including the development and modification of educational frameworks according to social requirements, evidence-based learning, and an increased emphasis on compassion and care among healthcare providers.^2^

Medical research should uphold ethical standards that guarantee respect for all human subjects while safeguarding their health and rights. Institutional Review Boards (IRBs) or Research Ethics Committees (RECs) are an integral part of research ethics. The main function of RECs is to conduct an impartial evaluation of research proposals to ascertain their adherence to ethical guidelines.^3^

Pakistan, classified as a low-middle income country and low literacy rates struggles with challenges stemming from insufficient investment in its healthcare system.^4^,^5^ In recent years many public and private sector medical colleges have established medical education departments, but currently Pakistan has 136 registered medical colleges, with 88 in the private sector. This rapid proliferation raises concerns about the quality of education being provided, as many teachers lack formal training in education and stick to traditional curriculum. Pakistan Medical and Dental Council (PMDC) mandated the establishment of medical education departments in all medical colleges in 2008, but no regulations or technical support was provided. This left many colleges struggling to establish and define the roles of these departments effectively.^2^ In Pakistan, there exists a two-tier system for ethics review, covering both institutional and national levels.

The COVID-19 pandemic and previously natural disasters like earthquake and floods have exposed the challenges faced by research ethics oversight in Pakistan, highlighting the urgent need for improved coordination and streamlined processes, especially for evaluating multicenter research projects. Despite efforts by the National Bioethics Committee-Research Ethics Committee (NBC-REC) to provide policy guidance for research, there has been minimal involvement of RECs/IRBs nationwide in this response. At the national level there is no program in place to review, register, scrutinize or inspect RECs/IRBs.^5^ Predatory publishing refers to journals that publish articles for a fee without rigorous peer review. They threaten the integrity of academic research by accepting bogus articles without proper peer review and prioritizing profit over quality. In Pakistan, where colonial roots influence educational policies, predatory publishing is investigated using Bourdieu’s concept of “symbolic violence”.^6^-^8^

There is no verifiable data from Pakistan indicating the exact number, nature, and capacity of ERCs, and there is no accreditation process to ensure standardization.^4^ This survey seeks to grasp what clinical research professionals in Pakistan think about the ethics of clinical research. To the best of our knowledge there is no previous study available. This study, the first of its kind in Pakistan, aims to pilot nationwide mapping exercises.

## METHODS

This was a prospective cross-sectional study conducted by the Department of Neurosurgery, Pakistan Institute of Neurosciences, Lahore, Pakistan in January and February 2024. This was a nationwide survey targeting residents of neurosciences. We used a non-probability, convenience sampling approach for selecting participants. The Institutional Review Board of Punjab Institute of Neurosciences, Lahore has issued an exemption letter for this web-based survey study, reference# 49/NS-I/2024.

Post-graduate residents of neurosciences who were doing their residency in Pakistan were recruited in this survey, irrespective of their age and gender. These included residents of neurosurgery and neurology with the degree programs of Fellow of College of Physicians and Surgeons (FCPS), Doctor of Medicine (MD) and Master in Surgery (MS). Post-graduate residents of other parent specialties who were on elective clinical rotations in the departments of neurosurgery and neurology were not included in this study.

We adapted a pre-existing general questionnaire in English, focusing on understanding attitudes and perceptions towards RE and RECs, to better suit local circumstances. The survey consisted of six sections. The introductory section provided an overview of the questionnaire’s purpose and included a declaration. Sections A, B, C, D, and E comprised both multiple choice questions (MCQs) and open-ended questions. The included sections contained relevant questions on the following topics (Table-I).

**Table-I:** Details of survey-questionnaire.

Sections	Areas covered
A	Demographic and professional details
B	The level of understanding and familiarity among medical postgraduates regarding research ethics and research ethics committees
C	Attitudes of medical postgraduates toward research ethics
D	Attitudes of medical postgraduates toward research ethics committees
E	Personal experiences

The survey utilized Google Form (Google Inc., Mountainview, CA) for a one-time sign-up response. It included an oath ensuring the anonymity of survey responses and pledging that post-graduate residents would respond voluntarily, honestly, and according to their own interest. Although more than 400 residents were approached by ambassadors, only 241 responded.

Ambassadors (data collectors) were hired from the provinces of Balochistan, Khyber Pakhtunkhwa, Punjab, Sindh and areas of Islamabad Capital Territory, Gilgit-Baltistan and Azad and Jammu Kashmir. There was also an oath taken by the ambassadors to ensure that they approach the target population only and ensure smooth recovery of responses with the will of participants. Ambassadors were instructed to remind the participants not more than three times in total and were allowed to contact as many post-graduate residents as possible according to the set criteria throughout Pakistan. Utilization of social media and close contacts was encouraged to disseminate the Form.

### Statistical Analysis:

Submitted responses were analyzed using the Statistical Package for Social Sciences (IBM SPSS Statistics for Windows, Version 24, released 2016; IBM Corp., Armonk, New York, United States). Analysis involved examining measures of central tendency, as well as calculating percentages and frequencies.

## RESULTS

**Fig.1:**
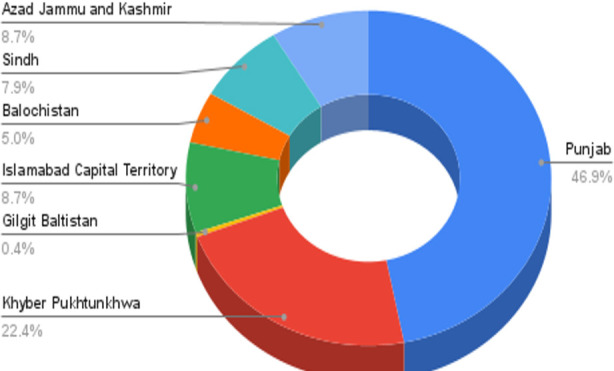
Provincial and regional distribution of respondents.

The majority of residents were from neurosurgery and among them 64.3% were male residents (Table-II). Majority of the participants were from Punjab followed by Khyber Pakhtunkhwa. A small proportion was also involved from Azad Jammu and Kashmir, Sindh, Islamabad and Gilgit Baltistan (Fig.1).

**Table-II:** Demographic and professional information of the participants, where N=241.

Item	Frequency, n	Percentage, %
***Gender***		
Male	155	64.3%
Female	86	35.7%
***Parent specialty***		
Neurosurgery	164	68%
Neurology	77	32%
***Year of residency program***		
I	64	26.6%
II	43	17.8%
III	37	15.4%
IV	24	10%
V	73	30.21%
***Training in research ethics***		
Yes	149	61.8%
No	132	54.8%

While more than half (51.5%) of our survey participants acknowledged the existence of an REC within their institution, and 52.3% were acquainted with ethical guidelines for research involving human subjects, a mere slight majority, specifically 48.5%, demonstrated awareness of the roles and responsibilities of RECs (Table-IV).

**Table-III:** Knowledge and awareness of postgraduate residents regarding research ethics and research ethics committee.

Sr. #	Questions	No %, n	Uncertain %, n	Yes %, n
1	To your knowledge, are there any official regulations or guidelines in Pakistan governing research ethics?	17.0% (41)	26.6% (64)	56.4% (136)
2	Does your hospital or institution offer a research ethics training program for postgraduates?	30.3% (73)	18.3% (44)	51.5% (124)
3	Does your degree-awarding body include a research ethics training program for postgraduates in your degree?	17.0% (41)	13.7% (33)	69.3% (167)
4	Does your university or hospital have a research ethics committee (REC)?	10.8% (26)	10.4% (25)	78.8% (190)
5	Do you believe that having a research ethics committee would be useful?	5.4% (13)	4.6% (11)	90.0% (217)
6	Are you aware of any committees or organizations that review the ethical aspects of research?	23.7% (57)	12.0% (29)	64.3% (155)
7	Did you attend workshops or lectures on research ethics?	30.7% (74)	1.7% (4)	67.6% (163)
8	Have you attended a course on research ethics or bioethics?	42.7% (103)	5.0% (12)	52.3% (126)
9	Do you have a comprehensive understanding of the functions of research ethics committees?	35.3% (85)	16.2% (39)	48.5% (117)
10	Are you acquainted with ethical guidelines governing research involving human subjects?	34.0% (82)	13.7% (33)	52.3% (126)
11	Have you heard of the Clinical Trial Registry of Pakistan?	54.8% (132)	7.9% (19)	37.3% (90)

**Table-IV:** Attitude of PGs toward research ethics.

Sr. #	Questions	Agree %, n	Disagree %, n	Neutral %, n	Strongly agree %, n	Strongly disagree %, n
1	Research ethics ought to be included as a mandatory module in postgraduate studies.	31.5% (76)	0.4% (1)	5.0 % (12)	63.1% (152)	0.0% (0)
2	All researchers should be trained in research ethics.	34.9% (84)	0.4% (1)	2.5% (6)	62.2% (150)	0.0% (0)
3	There’s a necessity for greater emphasis on research ethics when conducting studies involving human subjects.	30.7% (74)	0.85 % (2)	6.6% (16)	61.8% (149)	0.0% (0)
4	When involving patients in research with risks beyond the minimal level, it’s imperative to obtain informed consent from each patient.	27.4% (66)	2.9% (7)	3.7% (9)	66.0% (159)	0.0% (0)
5	When gathering data from research participants, precautions should be taken to avoid inadvertent disclosure of data.	27.0% (65)	0.0% (0)	7.9% (19)	64.7% (156)	0.4% (1)
6	If a blood sample is collected for clinical laboratory tests and the investigator wishes to utilize some of it for a research study, informed consent from the patient regarding the research study is not necessarily required.	18.3% (44)	33.2% (80)	10.8% (26)	18.7% (45)	19.1% (46)
7	When conducting clinical research, patients should not be informed about potential risks. Otherwise, they may not willingly agree to participate in the study.	18.3% (44)	33.2% (80)	10.8% (26)	18.7% (45)	19.1% (46)
8	Fabricating data or results to enhance research outcomes, when if there is no harm to patients, is acceptable.	16.2% (39)	27.0% (65)	16.2% (39)	11.25 (27)	29.5% (71)
9	It’s challenging to get a study published if the researcher fails to adhere to ethical guidelines.	41.5% (100)	10.0% (24)	19.9% (48)	26.1% (63)	2.5% (6)

A significant portion (44.5%) expressed the view that research evaluation by an ethics committee could hinder research advancement. Similar sentiments have been observed in previous studies, reflecting a collective concern regarding possible research delays stemming from REC scrutiny. Notably, our research unveiled that 37% of participants opted against disclosing potential risks to patients, apprehensive that it might discourage their involvement in the study. It’s interesting to see that about half (52.3%) of the respondents felt that informed consent should always be in writing. But it’s important to remember that informed consent should really focus on making sure people fully understand and feel comfortable, no matter how it’s documented. (Table-V).

**Table-V:** Attitude of post-graduate residents toward research ethics committees.

Sr. #	Questions	Agree %, n	Disagree %, n	Neutral %, n	Strongly agree %, n	Strongly disagree %, n
1	Every university or research institution should have a research ethics committee to conduct ethical reviews of research involving both humans and laboratory animals.	35.7% (86)	0.0% (0)	3.3% (8)	61.0% (147)	0.0% (0)
2	Research involving humans should undergo review by a research ethics committee.	33.6% (81)	0.4% (1)	3.3% (6)	63.5% (153)	0.0% (0)
3	Human research should undergo review by a research ethics committee prior to review by a scientific committee.	38.2% (92)	0.4% (1)	9.1% (22)	52.3% (126)	0.0% (0)
4	Ethical review contributes to enhancing the credibility of research.	40.2% (97)	0.4% (1)	7.9% (19)	51.5% (124)	0.0% (0)
5	Ethical review is solely for international collaborative research and projects.	21.6% (52)	33.6% (81)	19.1% (46)	17.4% (42)	8.3% (20)
6	Because there are scientific committees, research ethics committees are not necessary to review research.	16.2% (39)	45.6% (110)	14.9% (36)	12.9% (31)	10.4%(25)
7	Research ethics committee reviews may indeed cause delays and add complexity for researchers.	31.55 (76)	25.7% (62)	25.3% (61)	12.9% (31)	4.6% (11)
8	Members of research ethics committees should receive training in research ethics.	41.5% (100)	0.0% (0)	2.9% (7)	55.6% (134)	0.0% (0)
9	The members of the research ethics committee should ideally include professors or individuals with significant authority within universities.	36.1% (87)	8.3% (20)	22.0% (53)	33.2% (80)	0.4% (1)
10	To instill confidence in research ethics committee decisions, it’s essential for these committees to be subject to oversight from higher authorities.	51.0% (123)	1.7% (4)	14.5% (35)	32.0% (77)	0.8% (2)

In our study more than half of the participants were well aware of the ghost writing and a small proportion had already hired the services of a ghost writer. Among them 32% had not written the synopsis of their degree program themselves. This shows the malpractices and the need to educate and inculcate research ethics among residents (Table-VI).

**Table-VI:** Personal experiences of post-graduate residents.

Sr. #	Questions	Yes %, n	No %, n
1	Have you ever heard of "ghost writers"?	76.3% (184)	23.7% (57)
2	Have you ever hired the services of a ghost writer?	21.2% (51)	78.8% (190)
3	Is ghostwriting ethical to you?	12.9% (31)	87.1% (210)
4	Have you written the required synopsis/dissertation/thesis of your degree program yourself?	68% (164)	32% (77)

## DISCUSSION

The objective of this research was to evaluate and analyze the understanding, perspective, and behavior of neurosciences postgraduate residents regarding research ethics and the role of RECs. Through this survey, the study aimed to investigate the level of knowledge these residents possessed about research ethics principles, their attitudes towards ethical considerations, and their adherence to ethical practices in their research endeavors. Additionally, the discussion sought to identify any gaps or areas for improvement in the training and awareness of neurosciences postgraduate residents regarding research ethics, as well as the effectiveness of existing REC mechanisms in promoting ethical conduct within the field. Our study was the first study in Pakistan to include the residents of the Punjab Institute of Neurosciences in this regard.

Medical colleges need Departments of Medical Education for accreditation with PMDC. This reflects a global trend as more medical institutes establish these departments. It’s because people expect better healthcare, want accountability, and there’s a need to train more doctors due to growing healthcare demands.^2^

While there isn’t comprehensive empirical data to provide insights into the competencies of RECs/IRBs in Pakistan, existing publications have highlighted significant challenges. These challenges include lapses in ethics review practices, governance structure deficiencies, and shortages of trained personnel serving on RECs/IRBs. It’s important to note that these gaps are not exclusive to Pakistan.^5^

### Knowledge of research ethics and research ethics committees:

Understanding the knowledge, attitudes, and practices regarding our research topic is crucial both globally and at the national level for several reasons. Well-considered and comprehensive guidance for ethics review, adhered to throughout the process, can help steer a national approach in Pakistan.^4^ It helps ensure that research is conducted ethically and with integrity, which is essential for maintaining trust in scientific findings. Additionally, it ensures the protection of research participants and compliance with ethical regulations, promoting accountability and professionalism in research endeavors. As a result, it is imperative for medical schools to enhance their initiatives in creating and distributing courses and training programs focused on research ethics. These efforts should ensure that such training is compulsory and accessible to all medical postgraduates.^3^

Our questionnaire study marks the inaugural exploration from an academic establishment in Pakistan concerning the understanding, consciousness, and stances of post-graduates towards RE and RECs. Our findings indicate that although a notable proportion of post-graduates harbor various favorable attitudes towards the necessity for training in research ethics and the importance of research review by RECs, substantial disparities exist regarding their comprehension, awareness, and stances on research ethics and RECs. For instance, although over 51.5% of our respondents were aware of the presence of an REC within their institution and 52.3% were familiar with ethical guidelines for research on human subjects, only a slim majority i.e. 48.5% demonstrated awareness of the functions of RECs. Investigators have reported similar findings regarding awareness and knowledge of research ethics principles and the functions of RECs among faculty in Myanmar.^9^ In our study about 37.3% participants were aware of the existence of clinical trial registry of Pakistan compared to the participants of a study done in Lebanon in 2020 where only 27.4% were aware of the Lebanese National Consultative Committee on Ethics (LNCCE) and a study conducted in Central America and Dominican Republic clearly showed that only three countries (50%) have put in place formal written policies for health research and established official research priorities. Only in Costa Rica and Panama national RECs are legally mandated.^10^,^11^ Around 52.3% of the participants claimed that they have attended courses related to research and bioethics. In a single study, conducted in Pakistan in 2018, merely eight out of the total medical colleges (44.4%) reported having sufficient human resources for their Department of Medical Education (DME). Interestingly, it is worth noting an ironic twist: the personnel employed in DMEs lacked the requisite qualifications and training in medical education across all but one college.^2^

### Attitudes toward research ethics committees:

Concerning attitudes, there was a prevailing consensus regarding the necessity for an REC to conduct ethical reviews of research involving human subjects. However, a notable segment (44.5%) expressed the belief that research review by an ethics committee might impede the progress of research. Slightly greater percentages have been echoed in prior research, indicating a shared apprehension regarding potential delays in research due to REC review.^12^ The reasons underlying our respondents’ apprehensions regarding REC delays in research review could stem from their own unsatisfactory encounters with the REC approval process, coupled with a misunderstanding of the ethical review procedure. This is evident as fewer than a third of the respondents indicated awareness of the functions of RECs. To gain deeper insights into the perceived “delay” in research review, future investigations should include a comparative analysis of the turnaround time for research review by the country’s RECs against established benchmarks.

Our study revealed that 37% of participants preferred not to inform patients about potential risks, fearing it might deter their participation in the study. This sentiment closely aligns with findings from a study conducted at Jordan University of Science and Technology in April 2020, where 35.38% of participants shared similar perspectives.^13^ In our study, 27.4% of respondents agreed that fabricating research data to enhance research outcomes is acceptable. Given that this percentage is lesser than those reported in studies conducted in Western universities.^9^ However, it’s still reasonable to scrutinize the reliability of our findings regarding fabrication. Some might argue that our participants possibly misunderstood the concept of “fabrication.” However, it’s worth noting that our results are better than those obtained from investigations in other Low- and Middle-Income Countries (LMICs). For example, in a study involving researchers in Nigeria, the self-reported frequencies for falsification and fabrication were 27.5% and 29.8%, respectively.^9^

The present findings indicate a strong acknowledgment among respondents regarding the autonomy of research participants. However, when it comes to specific attitudes toward informed consent, there are discrepancies compared to the existing literature.^14^ Interestingly, approximately half (52.3%) of respondents expressed the belief that informed consent should always be in written form. However, it’s essential to recognize that informed consent should prioritize being both informed and culturally appropriate, irrespective of the method of documentation.

Mehta et al., in their eloquently written article published in May 2023, succinctly outlined the personnel, experiences, and responsibilities of ethical committee members. They highlighted that the composition of these committees varies depending on factors such as country, institution, research volume, and nature. Most importantly, at least one member must be autonomous and independent, with the chairperson being external to the research institution. Representation from the non-scientific community is essential for diverse perspectives in ethical decision-making.^15^ In our study, a substantial 69.3% of respondents advocated for research ethics committee members to be distinguished professors with significant authority within universities.

### Strengths and Limitations:

The sample size of 241 is a good number to generalize the results of this research for the state of affairs in Pakistan. Although, majority of the responses were from Punjab, it is important to note that most number of health-care centers of neurosciences are located in Punjab than any other province. Selection bias in Web-based surveys, unequal representation of neurosurgery and neurology residents, and lack of honest responses do not reflect the views of all resident trainees in Pakistan. To ensure honesty and integrity in this survey research, we introduced the concept of oath for honest, interested, consensual and anonymous replies.

## CONCLUSIONS

Our study found a notable correlation between participants’ attitudes towards research ethics and RECs and their understanding of ethics principles. These results suggest that medical postgraduates who possess greater familiarity or knowledge of research ethics principles and RECs are more likely to exhibit stronger attitudes towards these domains. Therefore, early education on ethics principles during their research careers can help students strike a balance between practical application and foundational research ethics principles, enabling them to navigate ethical dilemmas effectively in their future clinical research projects.

### Clinical Recommendations:

Here are actionable recommendations to strengthen research ethics and oversight:

1. Incorporate practical understanding of ethical standards among postgraduate residents by mandating a two-month rotation in research facilities or affiliations with medical journals.
2. Establish uniform regulations for medical ethics governance through active involvement of PMDC, promoting consistency and adherence throughout the healthcare sector to ensure consistency and effectiveness in ethical review processes.
3. Improve research standards by assigning each resident an editor from recognized PMDC journals to review their synopses, theses, and dissertations before acceptance. This will elevate the standard and quality of research output and the credibility of research findings.
4. Emphasize on medical ethics education into postgraduate training programs exit exams facilitated by researchers from PMDC-recognized journals.
5. Invest in training and capacity building programs for REC members to enhance their understanding of ethical principles, review processes, and regulatory requirements. This will improve the quality and efficiency of ethical reviews.
6. Promote transparency and accountability within RECs by establishing mechanisms for monitoring and evaluating their performance by including regular audits, feedback mechanisms, and public reporting of REC activities.
7. Encourage collaboration and networking among RECs, researchers, and regulatory bodies to facilitate information sharing, best practices dissemination, and mutual support in addressing ethical challenges.
8. Involve the public and community stakeholders in discussions and decision-making processes related to research ethics to ensure that research activities are aligned with societal values and priorities.

### Authors Contribution:

**HMQ** conceived and designed the study, analysed data and contributed to manuscript drafting and critical review.

**RS, SBF, NN, AK, SUH** collected data, interpreted results, did literature review and contributed to manuscriptt drafting.

**Collaborator Group** collected data, did literature review and contributed to manuscriptt drafting.

**ZMK** and **AB** supervised the study and critically reviewed the manuscript.

All authors approved the final version of manuscript.
